# Challenges in dietary management: a qualitative study on caregivers of preschool and school-age children with citrin deficiency

**DOI:** 10.1186/s13023-025-04099-z

**Published:** 2025-11-12

**Authors:** Shuxian Zhang, Lingli Cai, Xin Yang, Ni Gong, Qingran Lin

**Affiliations:** 1 National Clinical Research Center for Ocular Diseases, Eye Hospital, Wenzhou Medical University, Wenzhou, 325000 China; 2 School of Nursing, Jinan University, Guangzhou City, Guangdong Prov 510632 China; 3 The First Affiliated Hospital of Jinan University, Guangzhou City, Guangdong Prov 510630 China; 4 Department of Thoracic Surgery, Chongqing General Hospital, Chongqing, China

**Keywords:** Dietary management, Citrin deficiency, Caregivers, Children, School, Grounded theory

## Abstract

**Background:**

Reasonable dietary management is the most important and effective treatment for citrin deficiency. However, as this is a chronic and lifelong disease, managing citrin deficiency requires not only early diagnosis and the use of lactose-free or low-carbohydrate formula but also sustained adherence to a lifelong low-carbohydrate diet. This study aimed to investigate the dietary challenges faced by caregivers, particularly in non-familial settings such as schools, during children’s social development.

**Results:**

Seventeen participants were recruited for our study, comprising 16 mothers and 1 grandmother. We applied constructivist grounded theory to explore the dietary management challenges faced by caregivers. Four focused codes were developed through analysis of caregivers’ interviews: (1) Upon entering school, concealment is the best choice; (2) reality has rendered it unfeasible for me to maintain command over the situation; (3) teachers and peers are gradually participating in food management; and (4) children need to establish their own individual identities within society. The findings revealed that as children entered school, caregivers shifted from direct to indirect involvement in dietary management. The involvement of teachers and peers, along with the development of children’s autonomy, reduced the level of caregiver control over dietary management.

**Conclusions:**

After the children began school, the caregivers’ involvement in dietary management gradually decreased. The presence of teachers and peers complicates dietary management for preschool and school-age children with citrin deficiency, progressively diminishing caregiver control.

## Background

Citrin deficiency (CD) is an autosomal recessive disorder caused by biallelic mutations in SLC25A13, which located on chromosome 7q21.3 [1]. Common clinical manifestations include cholestatic jaundice, liver dysfunction, growth retardation, dyslipidemia, liver failure and other related symptoms [1–3]. At present, no specific drugs are available to treat citrin deficiency [4]. A high-protein, high-fat and low-carbohydrate diet is the most important and effective treatment for reducing liver damage in patients and improving clinical outcomes [5–7]. However, as a chronic and lifelong disease, the key to managing CD lies not only in early diagnosis and switching to lactose-free or low-carbohydrate formula, but also in fostering patient adherence to a lifelong low-carbohydrate diet. Non-adherence to this diet may lead to disease progression due to inappropriate dietary intake, resulting in liver failure, hyperammonaemic encephalopathy and other severe clinical manifestations [1, 8].

School enrolment significantly increases the frequency of social engagement and broadens the social experiences of children with CD, which diminishes caregivers’ direct control over dietary management and amplifies the complexity of management tasks. Yan et al. [9] conducted a survey on caregivers’ dietary management ability for children with CD, and the findings demonstrated that caregivers of school-aged children had less favourable dietary beliefs and were less aware of dietary risks than caregivers of non-school-aged children. Another study demonstrated a significant decline in the KAP scores of caregivers of children with CD after they were enrolled in school [10]. In contrast to the family-centred diet management model before school enrolment, the dietary intake of school-age children is influenced by various factors, including individual factors such as self-control and self-care abilities, interpersonal factors such as caregiver attitudes and peer pressure, and institutional or organisational factors such as school regulations [11, 12]. Promoting healthy eating in children requires cooperation from parents, educators, peers and other relevant stakeholders [11]. Furthermore, the quality of dietary management for school-age children relies on effective coordination between caregivers, teachers, peers and the children themselves. What dietary management challenges do caregivers encounter when their children start attending school? How do social and cultural factors affect caregivers’ dietary management behaviours?

Current research on the dietary treatment of CD primarily focuses on elucidating the pathophysiological mechanisms underlying low-carbohydrate diet therapy [13], determining the macronutrient composition ratio in patients’ diets [14], and investigating patients’ food preferences [15]. Limited research has been conducted on the dietary management challenges faced by caregivers of children with CD across different stages of growth, particularly during their school years. It is imperative to consider the contextualised dietary management of children with CD and further investigate the trajectory of changes in dietary management.

Therefore, to enhance the long-term efficacy of dietary management for children with CD, we investigated the dietary challenges faced by caregivers in non-familial settings alongside children’s social development.

## Methods

### Design

Constructivist grounded theory (CGT) is suitable for exploring issues that lack established theoretical frameworks [16]. Therefore, we adopted CGT to explore the dietary management problems encountered by caregivers after children with CD entered school. CGT requires researchers to keep an open mind during the research process and develop a theoretical framework based on data. Therefore, we entered the study without preconceptions, and the consolidate criteria for reporting qualitative research (COREQ) regulated our research presentation.

### Participants and sampling

A total of 17 participants were recruited from a tertiary hospital in Guangzhou, Guangdong Province, China. All participants were recruited via outpatient follow-up visits. The inclusion and exclusion criteria for participants are detailed in Table 1. We selected caregivers from different educational levels, occupations and regions to identify dietary management dilemmas in different contexts. Purposive and theoretical sampling were used to recruit participants to achieve maximum sample variation [17]. Following data saturation, the sample size was determined, and we stopped recruiting participants when additional data no longer contributed to theory development.

**Table 1:** The inclusion and exclusion criteria for participants

Inclusion and exclusion	
Inclusion	Have experience caring for school-age children with citrin deficiency
	Caregivers who are the primary dietary managers for children with citrin deficiency (including dietary management plan implementation and quality control)
	Have clear cognitive and expressive skills
	Provide formal informed consent to participate in the study
Exclusion	Caregivers who were employed by the family (e.g., hired nannies or nurses)

### Data collection

Seventeen semi-structured interviews were conducted between June 2021 and December 2022. The interview durations ranged from 24 to 106 min, with an average duration of 58 min. The first author conducted all interviews, and none of the participants were interviewed multiple times. Before each interview, we comprehensively explained the main purpose and content of the study to each participant. The participants could choose to participate voluntarily, and their decisions would not affect their children’s clinical treatment. They could indicate a preference for face-to-face or online interviews, depending on what was most convenient for them. No other relevant personnel were present during the interviews. All interviews were audio-recorded in real time and transcribed by the first author within 24 h of the interview. After the transcription, we sent the transcripts to the participants for confirmation. All researchers received professional training in core grounded theory principles, practising interview and observation techniques, as well as theoretical sampling and data management before data collection, and they had no prior contact with the participants. The semi-structured interview guidelines are presented in Table 2.

**Table 2:** Semi-structured interview guide

Questions
How have you managed your child’s diet since they joined school?
What obstacles have you encountered following changes in the dietary management environment?
How do teachers affect the dietary management of children?
How do peers affect the dietary management of children?
How do other parents affect the dietary management of children?
What is the impact of children’s autonomy development on dietary management?
What troubles you the most about dietary management at present?

### Data analysis

The data analysis was jointly performed by the first and corresponding authors simultaneously with the data collection. CGT provided important methodological support for data analysis. First, we encoded the transcript line-by-line without any preconceptions. We then integrated the initial codes based on their comparable meanings and features to generate the focused codes. Finally, we elucidated the relationships between various focused codes to generate broader conceptual categories (i.e. theoretical codes). The generated codes were discussed once a week through a discussion group (comprising three female nursing postgraduates, one female clinical nurse, and one male anthropologist), which helped us mitigate researcher bias, enhance theoretical sensitivity, and verify analytical consistency during our study. At each data analysis stage, we employed a constant comparative method to determine how the theoretical framework fit the broader context of the study. Additionally, reflective memos were written throughout the research process and used to assist in collecting more information to enrich the theoretical framework.

### Ethical considerations

This study was approved by the medical ethics committee (KY-2022-052). All participants signed informed consent forms before the study commenced. No third party could access the data other than the study team.

## Results

A total of 17 participants were recruited in our study. The descriptive characteristics of the participants are presented in Table 3. Using CGT, 1theoretical codes, 4 focused codes and 12 initial codes were constructed (see Table 4). The findings indicated a gradual decline in the caregivers’ involvement in dietary management after the children commenced schooling, whereas the participation of teachers, peers, and children increased.

**Table 3:** Descriptive characteristics of caregivers (*n* = 17)

Characteristic	$\:\stackrel{-}{\varvec{X}}$±SD / *n* (%)
Age	34.71 ± 8.64
Gender	
Female	17(100%)
Male	0
Relationship with patient	
Mother	16(94.12%)
Grandmother	1(5.88%)
Educational level	
Primary school	1 (5.88%)
Junior high school	4 (23.53%)
Senior or vocational high school	5 (29.41%)
College degree or above	7 (41.18%)
Employment	
Yes	12 (70.59%)
No	5 (29.41%)
Place of residence	
East China	2 (11.76%)
South China	9 (52.94%)
Central China	4 (23.53%)
Southwest China	2 (11.76%)
Having religious beliefs	
Yes	2 (11.76%)
No	15 (88.24%)

**Table 4:** Theoretical codes, focused codes and initial codes

Theoretical codes: Transfer of dietary control in home-school conflict	
Focused codes	Initial codes
Upon entering school, concealment is the best choice	I do not want my child to experience unfairness
	I cannot damage my child’s public image
	I need to consider issues from the perspectives of other parents and teachers
Reality has rendered it unfeasible for me to maintain command over the situation	I cannot change the school’s dietary structure
	I cannot change the school’s dietary management regulations
	The direct supervision of my child is reducing
Teachers and peers are gradually participating in food management	Teachers do not understand the importance of special diets
	Teachers disregard dietary management requirements
	The eating behaviours of classmates stimulate children’s curiosity
Children need to establish their own individual identities within society	I will not change my child’s social environment
	I hope my child can solve problems by themselves
	Children’s autonomous behaviour is becoming increasingly apparent

### Upon entering school, concealment is the best choice

This category elucidates the caregivers’ decision-making process regarding dietary management amid the significant environmental changes resulting from enrolment. The primary motivations for caregivers to conceal children’s dietary needs were to prevent potential discrimination, protect the children’s public image, and consider issues from others’ perspectives.

### I do not want my child to experience unfairness

Although hepatic metabolic diseases are noncontagious, most people are wary of liver diseases. The caregivers said that many individuals tended to unknowingly associate liver disease with ‘infection’ unknowingly. Consequently, to prevent potential misinterpretations from teachers, they concealed their children’s ailments to ensure equitable access to education.

*I’m concerned about others’ potential perception of contagion associated with the child’s illness*. (caregiver No.7)

*I was hesitant to disclose the truth to the preschool owing to concerns that they might reject my child*. (caregiver No.13)

Additionally, the psychological well-being of children was a matter of concern for caregivers. They worried that teachers and classmates would discriminate against their children. This dilemma also presented caregivers with the challenge of balancing their children’s dietary management with their mental well-being.

*I don’t want them to think my child is unhealthy. I’m afraid that his teachers and peers may regard him with a peculiar countenance*. (caregiver No.7)

### I cannot damage my child’s public image

When children lack sufficient self-expression skills, caregivers assume the role of facilitators in shaping their identity. Some caregivers were reluctant to disclose their child’s special dietary needs, not because of a lack of appreciation for dietary management but because of concerns regarding the potential adverse effects of disease stigma on their child. They aimed to prevent their child’s illness from becoming a subject of discussion during social gatherings.

*I’m afraid that people with questionable ethics will potentially spread information about the illness extensively*. (caregiver No.10)

*After all, it’s a gene-related disease, and it can’t be cured immediately. I’m afraid others will spread rumours*. (caregiver No.14)

Furthermore, the caregivers were concerned that disclosing their children’s illness would have a detrimental impact on their prospects. They believed that undesirable and unrealistic rumours may affect their children’s lives or even their eventual marital status.

*Others knowing about the genetic defect is not good for his future life and may diminish his prospects of forming relationships and starting a family*. (caregiver No.15)

### I need to consider issues from the perspectives of other parents and teachers

When discussing potential discrimination, the caregivers asserted that their suspicions were not entirely baseless. Prior to their children’s diagnosis, they also lacked the knowledge to ascertain the impact of CD on others’ health. Furthermore, for others, the impact of CD on their health was not deemed paramount. In their perspectives, a clear dichotomy existed between ‘illness’ and ‘health’. The caregivers said that they also would not want their child to grow up with other ‘unhealthy’ children.

*If my child were healthy and there was a special child in the class, I might also have concerns*. (caregiver No.13)

Moreover, some caregivers were hesitant to disclose the truth to teachers because of worries that the teachers may not have the energy to provide special dietary services to their children.

*I also work in a preschool, and I know that teachers don’t have that much energy. (caregiver No.9)*

With so many students in a class, it becomes impractical for teachers to effectively attend to each student. If I talk about this too much with teachers, it will be more stressful for them to take care of my child. (caregiver No.12)

### Reality has rendered it unfeasible for me to maintain command over the situation

This category elucidates the caregiver’s diminishing control over children’s dietary management upon school enrolment, including the inability to change the school’s dietary structure and management regulations, as well as a progressively reduced duration of directly supervising children.

### I cannot change the school’s dietary structure

The type of diet provided by schools is crucial in determining the quality of children’s dietary management during schooling. Under the influence of traditional dietary structures, the meals provided by schools also have a high-carbohydrate profile. Some caregivers reported that the limited high-protein food provided by the school could not effectively meet the nutritional needs of the children because of the lower food allowance paid by the parents.

Breakfasts provided by the school are usually carbohydrate-based, such as noodles and porridge. And afternoon snacks are also mostly sweet, such as steamed buns and cakes. (caregiver No.13)

Because the school receives only a limited amount of money for meals, the amount of meat provided is also limited. (caregiver No.10)

Furthermore, most schools have a centralised food management system, leaving children with no choice but to accept the food provided by the school. The caregivers stated that the school would not provide special dietary services for their children and that preparing separate meals for them was not feasible.

At school, all students receive the same meals, and it is impossible to prepare a separate meal just because my child doesn’t like the dish. (caregiver No.9)

### I cannot change the school’s dietary management regulations

Preparing homemade lunches for children is an effective way for caregivers to improve their children’s dietary intake at school. However, most schools are not only unable to provide special dietary services for children with CD but also do not allow them to bring their own meals. The caregivers said that, to prevent food poisoning and other regrettable situations, all students who eat on campus must consume food cooked by the school cafeteria.

For safety reasons, the school doesn’t allow us to bring any food. The school doesn’t want to take any extra risk. (caregiver No.16)

While some schools may have previously accommodated children by allowing them to bring their own meals, most schools implemented restrictions during the COVID-19 pandemic to minimise the risk of virus transmission. Consequently, regardless of their dietary preferences, the children were required to consume meals provided by the school.

During the pandemic, kids were not allowed to go home for lunch. They had to eat at school.(caregiver No.1)

He had lunch at the school cafeteria for a month during the pandemic. At that time, students were not allowed to go home for meals. (caregiver No.6)

### The direct supervision of my child is reducing

After schooling began, the frequency of interactions between the caregivers and their children diminished. The caregivers could no longer monitor their children’s food consumption directly or regulate their carbohydrate intake during school hours. Time constraints placed the caregivers in a passive role in managing their children’s diets, gradually diminishing their absolute authority over dietary management.

I know which food should not be eaten in excess. I can control my child’s intake at home, but it’s impossible to do the same at the preschool. (caregiver No.13)

Therefore, the caregivers routinely communicated with children to gain an understanding of their dietary intake during school. Nevertheless, most children lacked the ability to provide accurate reports on their dietary intake, posing challenges for the caregivers in verifying the accuracy of their reports.

I will ask my son what he ate at preschool, but he can’t tell me clearly. (caregiver No.2)

He will tell me what he ate when he comes back from school, but I really don’t know whether he ate it or how much he ate. (caregiver No.15)

### Teachers and peers are gradually participating in food management

This category delineates the influence of teachers’ and peers’ behaviours on the efficacy of school meal management for children with CD. Teachers’ inadequate understanding of dietary management and disregard for caregivers’ requirements and peers’ unhealthy eating behaviours were found to be significant impediments to the successful implementation of dietary management efforts.

### Teachers do not understand the importance of special diets

People’s awareness of CD is limited, and most teachers lack familiarity with its dietary principles. The caregivers reported that, owing to a lack of disease awareness, teachers struggled to comprehend specific dietary requirements and failed to recognise the importance of limiting carbohydrate intake.

Teachers have never heard of CD, and they have never heard that eating too much rice is harmful. (caregiver No.15)

If the teacher is not particularly responsible, she wouldn’t bother learning about this disease or understand the importance of dietary management. (caregiver No.9)

Furthermore, from the perspective of some teachers, children with CD may not seem significantly different. The teachers’ initial impressions of these children appear indistinguishable from those of their peers. They are equally lively and active.

In the teacher’s eyes, he is just as lively and energetic as his peers. (caregiver No.9)

From the outside, you can’t tell the difference between her and the other kids because she is very lively. (caregiver No.13)

### Teachers disregard dietary management requirements

Owing to an insufficient understanding of the disease, some teachers may not consider the importance of dietary management from caregiver’s perspective and may even oppose caregiver’s dietary requirements. They may attribute children’s picky eating behaviour to their caregivers’ excessive indulgence.

I told the teacher that his situation was special. If he ate too much rice, he would feel uncomfortable. The teacher said it was a case of picky eating and needed to be corrected gradually. (caregiver No.8)

I explained to the teacher patiently, but the teacher thought that I was just trying to make my child eat better at school and believed that I was unreasonable. (caregiver No.15)

Furthermore, some teachers were concerned about whether the children had completed the tasks assigned to them by the schools. They required children with CD to participate in the ‘Clear Your Plate’ campaign like other students, and if the child failed to complete the task, they would be punished.

She happily told me: “I finished all the rice today.” I suspected that the teacher encouraged them to finish their rice, and she felt happy that she had done it. But I would rather she hadn’t eaten it. (caregiver No.13)

She told me that the teacher doesn’t allow them to waste rice, and if they don’t finish it, they will be punished. (caregiver No.14)

### The eating behaviours of classmates stimulate children’s curiosity

Classmates represent one of the primary social groups of children, who regularly interact with them upon entering school. Their dietary preferences may subtly affect their eating habits. The caregivers reported that their children had become curious about and interested in the food enjoyed by their peers.

She also wants to eat what she sees someone else eating. (caregiver No.1)

If he sees many classmates eating a certain kind of snack, he also wants to try it. (caregiver No.7)

Confronted with children’s inquisitiveness and anticipation, some classmates altruistically shared their provisions. The caregivers said that they would usually not ask their children to refuse food offered by other classmates because sharing is widely regarded as a traditional virtue by caregivers who are keen on children not spurning acts of kindness because of their illness.

I told him that if someone shares their food with him, he can take it, but he should bring it home and ask me if it’s okay to eat. (caregiver No.11)

### Children need to establish their own individual identities within society

This category delineates the challenges caregivers face in dietary management during their children’s socialisation. Maintaining a healthy social environment and nurturing children’s problem-solving abilities and autonomous behaviour were found to increase the difficulty of dietary management.

### I will not change my child’s social environment

Children with CD encounter greater difficulties and obstacles in social interactions. Some caregivers were reluctant to disclose their children’s dietary restrictions to other parents when their children visited their classmates’ homes. This hesitation stems from a lack of familiarity with the characteristics of other parents and a desire to avoid potential misunderstandings that could hinder their children’s social opportunities.

Many people don’t know about CD. And in this society, there are all kinds of parents. If they knew my child had a disease, they might not want their child to play with mine. (caregiver No.14)

The caregivers stated that they would not deliberately change their children’s social environment as they prioritised enjoyable social interactions. They thought supporting their children in preserving a positive psychological state and fostering constructive interpersonal relationships was important.

I think it’s more important to be happy… I don’t want other classmates to say mean things to her. (caregiver No.14)

Compared with dietary management, I think the growth environment is more important. (caregiver No.13)

#### I hope my child can solve problems by themselves

To minimise the disruption of children’s social environments, some caregivers allowed their children to independently navigate dietary management issues during social interactions. The caregivers said that rice and other high-carbohydrate foods posed psychological barriers for their children when they visited their classmates’ homes. However, owing to their limited social skills, many children were afraid of expressing their specific dietary needs when they encountered the kindness of other parents.

They (classmates’ parents) would serve her rice. But she would feel a lot of pressure when she saw a full bowl of rice because she knew she couldn’t eat much rice and was afraid to say so. (caregiver No.14)

Despite being aware of the challenging circumstances faced by children, the caregivers still refrained from directly intervening in their social lives as they believed that children are best suited to communicate their specific dietary needs and must learn to manage these issues.

Most of the time, I will let her express the need by herself because I can’t always be with her. (caregiver No.16)

I can provide protection for him in his youth. But as he grows up, he must learn to protect himself. It is not feasible for me to shield him indefinitely. (caregiver No.15)

### Children’s autonomous behaviour is becoming increasingly apparent

Meanwhile, the increasingly autonomous behaviour of children tended to posed challenges for the caregivers in managing their dietary intake. Despite the caregivers’ efforts to restrict unsuitable food consumption for disease management, children who did not fully understand the significance of dietary control would engage in unsupervised eating.

He will buy the snacks clandestinely, which he didn’t do before. (caregiver No.8)

I have told him which foods he shouldn’t eat too much of, but sometimes he will be sneaky and eat them out of my sight. (caregiver No.7)

Moreover, as children enhance their decision-making skills, they gradually begin to consider the advantages and disadvantages of their dietary management processes. Children who do not believe in the importance of dietary management may opt to temporarily forgo it.

They have their own individual scores at school, and any food wastage will result in a deduction of points by the teacher. If her points are deducted for wasting food, she will not be happy, so she always finishes all the rice. (caregiver No.16)

## Discussion

This study employed CGT to investigate the challenges caregivers face in managing their children’s diet after they begin school and to better understand how caregivers manage diets outside the familial setting. The findings indicated a gradual decline in caregivers’ involvement in dietary management after the children commenced schooling and an increase in participation from teachers, peers and the children. Drastic changes in the dietary management environment have undermined caregivers’ direct control of their dietary management work. Therefore, whether caregivers can align with teachers and other stakeholders regarding dietary management will profoundly impact the quality of dietary management for school-age children with CD. However, addressing differences in understanding between caregivers and other stakeholders depends not only on whether caregivers are willing to proactively disclose the child’s medical condition but also on how well other dietary management participants understand the importance of special diet principles.

Proactive disease disclosure by caregivers is essential for effective management of CD by other participants [18]. However, our study demonstrated that many caregivers were not willing to inform school personnel about the special dietary requirements of children and regarded concealment as the best option. Fearing discrimination, many caregivers choose not to disclose their child’s condition to protect their educational opportunities, future development, and even marital prospects. The fear that children may be denied equal educational opportunities serves as the primary motivation for caregivers to conceal their children’s conditions during the early stages of schooling. They were uncertain about how teachers would treat their children after disclosure and whether classmates would be willing to befriend their children. Ultimately, caregivers’ adherence to dietary management is affected by their desire to avoid potential negative consequences for children [19].

In addition, caregivers found it difficult to maintain control over their children’s diets after they entered school. Providing school meals that meet special dietary requirements is essential for maintaining consistent and effective dietary management for children with CD during school hours. Similar to the traditional high-carbohydrate diets prevalent in East Asia [20], school meals tend to be rich in carbohydrates. Furthermore, most schools face challenges in offering personalised meals to children, making it difficult for them to meet their specific dietary needs during school hours. And consider of dietary safety, nearly all schools prohibit caregivers from preparing homemade food. School regulations further hinder children’s ability to receive proper nutrition [21]. Another obstacle is the reduced ability of caregivers to directly supervise their children’s dietary habits. They face challenges in gaining timely and accurate insights into their children’s dietary intake during school hours. Children’s activities outside the home often make it difficult for caregivers to ensure consistency in the provision of healthy meals [22].

The growing involvement of teachers and peers has complicated dietary management. Due to traditional diet structures, many teachers lack an understanding of the crucial role of a low-carbohydrate diet in disease management in children with CD. They may not recognise rice as a potential causative factor and may perceive selective eating behaviour in children with CD as simply a bad habit requiring correction. Teachers’ cognition and comprehension of dietary management plans significantly influence their implementation [23, 24]. Consequently, some teachers still enforce high-carbohydrate diets for children with CD. Their inadequate awareness of proper dietary management is a key reason for school-age children’s deviation from their prescribed dietary plans [21]. Additionally, peers’ eating behaviour also exerts a significant influence on children’s daily dietary intake [25, 26]. Curiosity about their peers’ diets and a desire to fit in often lead children to make independent food choices that conflict with their dietary restrictions [27]. However, owing to their limited self-control and self-care skills, most children struggle to manage their diets effectively without parental supervision [12].

Ultimately, the caregivers wished that they could help their children establish their own individual identities within society. Many caregivers said that in addition to short-term concerns, they also considered the long-term implications of disclosing their children’s diseases. They wanted to preserve their children’s social environment, strived to prevent others’ misconceptions or negative perceptions about their children and aimed to safeguard their children’s future career prospects and relationships. Long-term negative repercussions associated with disease management can undermine caregivers’ efforts to manage the disease effectively [28]. Additionally, the caregivers recognised that navigating dietary management challenges within social contexts is an essential skill for school-age children during their socialisation processes. When caring for children with special dietary requirements, most caregivers encourage their children to become self-reliant in dietary management [29]. Consequently, they refrain from direct involvement in dietary management problems encountered by children during social processes. They start weighing the pros and cons of following their diet and thinking about how food affects their social interactions [30]. The conflict between personal interests and disease management measures is also a crucial factor that challenges children’s commitment to dietary management.

In conclusion, the successful transition from home-based to school-based dietary management for children with CD depends on caregivers’ ability to work effectively with teachers and other school staff to implement appropriate dietary principles. To enhance the quality of dietary management for children with CD in school, healthcare professionals must assist caregivers in recognising the significance of proactive disease disclosure and foster an understanding of specialised dietary principles among teachers and other participants involved in dietary management. Furthermore, medical personnel may facilitate a cooperative relationship between caregivers and other participants in dietary management by providing scientific and professional educational materials, such as informative brochures and popular science videos.

### Recommendations

Following are the recommendations for caregivers. (1) They must clearly articulate their children’s specific dietary management requirements to school personnel. 2) They must ensure timely communication with children and assess dietary intake during school hours. 3) They must teach children essential dietary principles and foster autonomy in making food choices.

Following are the recommendations for teachers. (1) They must objectively and rationally understand parental requests for specialised dietary management of children. (2) They must accurately implement specialised dietary management requirements and maintain dynamic communication with parents. (3) They must ensure equitable treatment for all students with special dietary needs.

Following are the recommendations for institutions and schools. (1) They must permit students with special dietary requirements to bring meals from home. (2) They must provide specialized diets to children with special dietary requirements. (3) They must involve professional nutritionists in collaboration with teachers to manage students’ special dietary requirements.

### Limitations

This study was conducted within the specific dietary and educational context of China, and its applicability to other countries warrants further investigation. The challenges caregivers face in dietary management may vary across cultural contexts. However, the fundamental reasoning for resolving school dietary management problems remains the same. Nevertheless, this study offers valuable insights into the caregiver perspective. Teachers and other participants in dietary management were excluded from this study. To obtain more comprehensive research findings, this study should be expanded to include additional participants in dietary management.

## Conclusion

This study identified the challenges caregivers face in managing their children’s diets after they start school. The presence of teachers, peers, and other individuals involved in dietary decisions posed significant challenges to the caregivers’ management efforts. Addressing differing views on dietary management among school stakeholders is essential to enhancing the quality of diet management at school. Furthermore, these findings provide a reference for how caregivers can better adapt to changes in dietary management patterns and improve the long-term efficacy of dietary management for CD.

## Data Availability

The data presented in this study are available on request from the corresponding author. The data are not publicly available due to [restrictions e.g., their containing information that could compromise the privacy of research participants].

## Abbreviations

CD: Citrin deficiency
CGT: Constructivist grounded theory
COREQ: Consolidated criteria for reporting qualitative research
